# Peroxidase-like Active Cu-MOFs Nanozymes for Colorimetric Detection of Total Antioxidant Capacity in Fruits and Vegetables

**DOI:** 10.3390/foods14081311

**Published:** 2025-04-09

**Authors:** Yanyan Huang, Jiatong Han, Yi Ping, Xin Chen, Yiming Zhao, Ge Chen, Jun Lv, Donghui Xu, Yanguo Zhang, Jing Chen, Guangyang Liu

**Affiliations:** 1 State Key Laboratory of Vegetable Biobreeding, Institute of Vegetables and Flowers, Chinese Academy of Agricultural Sciences, Key Laboratory of Vegetables Quality and Safety Control, Ministry of Agriculture and Rural Affairs of China, Beijing 100081, China; huangyanyan0412@163.com (Y.H.); han.jiatong@outlook.com (J.H.); pingyi0819@163.com (Y.P.); chenxin2721@163.com (X.C.); kazuakizhao@163.com (Y.Z.); chenge@caas.cn (G.C.); lvjun02@caas.cn (J.L.); xudonghui@caas.cn (D.X.); zhangyanguo@caas.cn (Y.Z.); chenjing_nt@163.com (J.C.); 2 National Center of Technology Innovation for Comprehensive Utilization of Saline-Alkali Land, 8 Zhihui Road, Agricultural High Tech Industry Demonstration Zone, Yellow River Delta, Dongying 257347, China

**Keywords:** Cu-MOFs, nanozyme, peroxidase, fruits and vegetables, total antioxidant capacity

## Abstract

In this study, two types of Cu-MOFs (Cu-TCPP and CuO-TCPP) with a two-dimensional layered porous structure were prepared via in situ polymerization using Cu^2+^, CuO, and TCPP as raw materials. Both Cu-MOFs exhibited peroxidase-like activity, capable of catalyzing the oxidation of TMB by H_2_O_2_ to form oxTMB, resulting in an absorption peak at 652 nm and a color change from colorless to blue. Subsequently, the addition of AA can reduce oxTMB back to TMB, causing the color of the system to lighten or become colorless. Based on this principle, a simple and rapid colorimetric method for AA detection was established and successfully applied to the detection of TAC in fruits and vegetables. The results showed that Cu-TCPP and CuO-TCPP had a large linear range of ascorbic acid detection of 0.01–100 mM (Cu-TCPP) and 0.05–100 mM (CuO-TCPP). This study not only provides a novel method for preparing nanozymes with peroxidase-like activity, but also offers a simple approach for analyzing the TAC of food.

## 1. Introduction

Antioxidants are a type of substances capable of delaying or preventing oxidative reactions [1], serving as essential substances for maintaining the balance of reactive oxygen species [2]. Antioxidants come in a wide range of varieties, including vitamin C, vitamin E, β-carotene, glutathione, and polyphenolic compounds [3,4]. These substances are widely found in various natural foods, such as fruits, vegetables, nuts, whole grains, and tea [5,6,7]. Antioxidants play a significant role in maintaining human health. They can delay cell senescence [8]; maintain skin elasticity and gloss [9,10]; reduce the risk of chronic diseases such as renal diseases [11,12], cardiovascular diseases [13,14,15], inflammation [16,17], and cancer [18,19]; enhance immunity to resist viruses and bacteria [20]; and promote the health of the nervous system, thereby improving memory and cognitive ability [21]. Therefore, it is necessary to evaluate the antioxidant capacity of different foods for people’s healthy diets.

Currently, methods for detecting total antioxidant capacity (TAC) include Oxygen Radical Absorbance Capacity (ORAC), Ferric ion Reducing Antioxidant Parameter (FRAP), [2,2′-azinobis(3-ethylbenzothiazoline-6-sulphonic acid)] (ABTS), voltammetric determination, 2,2-diphenyl-1-picrylhydrazyl (DPPH), Trolox Equivalent Antioxidant Capacity (TEAC), Folin-Ciocalteu methods, Cupric Reducing Antioxidant Capacity (CUPRAC), ferricyanide/Prussian blue assay, and others [22,23,24,25,26,27,28,29,30]. However, these methods are often hampered by the limitations of natural enzymes [31], the high cost of equipment, and the cumbersome sample processing procedures. In contrast, colorimetric methods have been widely used in the field detection of food components due to their advantages, such as simple readout, convenient operation, low cost, and the high reliability of measurement results [32,33]. Among them, colorimetric methods relying on enzyme-catalyzed chromogenic substrates are one of the most common types [34]. Nevertheless, natural enzymes have several drawbacks, including high costs for preparation, purification, and storage, strict reaction conditions required, and time-consuming production processes [35,36,37]. These factors hinder their widespread application in colorimetric detection methods.

In recent years, with the rapid development of nanotechnology, artificial enzymes based on nanomaterials possess the “dual characteristics” of enzymes and nanomaterials, offering advantages such as facile synthesis, high stability, and a relatively low cost, making them potential alternatives to natural enzymes [38,39,40]. So far, various materials have been utilized for the preparation of nanozymes, including carbon-based nanomaterials [41,42], noble metals [43], covalent organic frameworks [44], and metal-organic frameworks [45]. These nanozymes exhibit diverse redox enzyme activities, such as oxidase [46,47], catalase [48,49], peroxidase [50,51], and superoxide dismutase [42,52], and have been applied in fields such as environmental protection [53,54], biomedicine [55,56], and the food industry [57,58]. In recent times, there have been extensive reports on TAC detection methods based on peroxidase activity [59,60,61,62,63]. While most research focuses on carbon-based nanomaterials and monometallic materials, the application of metal-organic frameworks (MOFs) in the detection of TAC remains scarce, and most practical samples studied are commercial beverages and medical vitamin C tablets.

MOFs have garnered significant attention due to their advantages, such as a large specific surface area, high porosity, adjustable structure, and an inorganic–organic hybrid structure that can be designed to possess diverse mimetic enzyme activities [64]. The ultrathin structure and large surface area of two-dimensional MOFs facilitate the exposure of a large number of active sites, thereby enhancing their catalytic activity and offering broader application prospects [65].

Compared to precious metals such as Au and Ag, Cu is an indispensable trace element in the human body, serving as an integral component of numerous natural enzymes. Furthermore, Cu and Cu-based nanomaterials boast the advantages of low cost and high natural abundance [66,67]. Consequently, a variety of Cu-based nanomaterials, including copper oxide, Cu metal-organic frameworks, and CuS, have been developed and have demonstrated considerable potential in the field of enzymatic catalysis [68]. In this study, two copper-based MOFs nanomaterials (Cu-TCPP and CuO-TCPP) with peroxidase-like activity were prepared by using the original polymerization method. The preparation methods of these two nanozymes are straightforward, and they possess good thermal stability and peroxidase-like activity (Cu-TCPP and CuO-TCPP have Km values of 0.27 mM and 0.57 mM, respectively). These nanozymes can be utilized for the detection of ascorbic acid (AA) and TAC.

## 2. Experimental Section

### 2.1. Materials and Instruments

3,3′,5,5′-tetramethylbenzidine (TMB) was purchased from Shanghai Aladdin Biochemical Technology Co., Ltd. (Shanghai, China). L-Ascorbic acid was purchased from Xilong Chemical Co., Ltd. (Xilong, China). Cupric sulfate was purchased from Beijing Chemical Works (Beijing, China). Sodium citrate was purchased from SuZhou Ruri Chemical Technology Co., Ltd. (Suzhou, China). Sodium borohydride was purchased from Jiangsu Runfeng Synthesis Technology Co., Ltd. (Nantong, China). N, N-Dimethylforma mide and 4,4,4,4-(Porphine-5,10,15,20-tetrayl) were purchased from Shanghai Macklin Biochemical Technology Co., Ltd. (Shanghai, China). Ethanol, Benzoic acid, Cupric chloride, Cupric Acetate Monohydrate, and Acetic Acid were bought from Sinopharm Chemical Reagent Co., Ltd. (Shanghai, China). Chinese cabbage, kiwi, orange, and cabbage all were purchased from local supermarkets. For this study, we employed a NanoDropOne UV-Vis spectrophotometer to measure the absorbance.

### 2.2. Preparation of Nanozyme

The synthesis methods of Cu-TCPP and CuO-TCPP refer to research by Duong Duc La et al. [69] and improve the synthesis by in situ polymerization. Firstly, a 2.0 mL 1.0 mmol/L meso-Tetra (4-carboxyphenyl) porphine (TCPP) suspension using N,N-Dimethylformamide (DMF) was configured. After the complete dissolution of TCPP, 1.0 mL of 10 mM CuSO_4_, CuO aqueous solutions were added separately into a 100 mL round-bottom flask, and then 0.18 g of benzoic acid and 10 mL of DMF were added, and the mixture was magnetically stirred at 90 °C for 4 h. After cooling to room temperature, the mixture was centrifuged at a speed of 9000 r/min for 15 min, and then the precipitate obtained was washed after centrifugation three times with deionized water/ethanol. Finally, it was freeze-dried for 12 h, and the resulting purple and dark purple powder-like solids, which are Cu-TCPP and CuO-TCPP, were ground and collected.

### 2.3. Characterization

Scanning Electron Microscopy (SEM) was utilized for characterizing particle size, morphological features, and compositional analysis. The ASAP 2020 analyzer was employed to determine properties such as the Brunauer-Emmett-Teller (BET) surface area and N_2_ adsorption–desorption isotherms. Crystal structure analysis was conducted using an advanced X-ray powder diffractometer (XRD). The Fourier-transform infrared spectroscopy (FTIR) spectra of the synthesized nanomaterials were recorded using a Fourier spectrophotometer. An X-ray photoelectron spectroscopy (XPS) instrument was utilized to measure the X-ray photoelectron spectroscopy maps. The thermal stability of the materials was assessed using a NETZSCH STA 449 F3/F5 thermal analyzer.

### 2.4. Peroxide-like Activity of Cu-TCPP and CuO-TCPP

The peroxidase-like activity of Cu-TCPP and CuO-TCPP was studied using the chromogenic reaction between H_2_O_2_ and TMB. Firstly, TMB (15 mM, 0.1 mL) and H_2_O_2_ (30%, 0.1 mL) solution were added to acetate buffer solution (10 mM, 3 mL, pH = 4). And then an aqueous solution of Cu-TCPP or CuO-TCPP (1.5 mg/mL, 0.1 mL) was added, and it reacted at room temperature for 15 min. Color changes were observed, and the absorbance of the reaction system was measured using UV-visible spectroscopy at a wavelength of 652 nm. Firstly, a systematic study was conducted to investigate the effects of nanozyme concentration, reaction time, TMB concentration, and H_2_O_2_ volume on enzymatic activity. And then, through seven sets of comparative experiments (Nanozyme + TMB + H_2_O_2_, Nanozyme + H_2_O_2_, Nanozyme + TMB, TMB + H_2_O_2_, TMB, Nanozyme, H_2_O_2_), the peroxidase activity of Cu-TCPP and CuO-TCPP was verified.

Secondly, the steady-state dynamics of Cu-TCPP and CuO-TCPP were analyzed. Aqueous solution of nanozyme (1.5 mg/mL, 0.1 mL), acetate buffer solution (3 mL), H_2_O_2_ solution (0.1 mL), and different concentrations (0–2 mM) of TMB solution were used to determine steady-state dynamics. The kinetic constants are derived through the Michaellis-–Menten equation:

$$\frac{1}{v}=\frac{K_{m}}{V_{max}}\times \frac{1}{\left[S\right]}+\frac{1}{V_{max}}$$

In this equation, *v* represents the initial reaction rate, *V_max_* is the maximum reaction rate, *K_m_* is the Michaellis–Menten constant, and [*S*] represents the substrate concentration.

### 2.5. Colorimetric Detection of Antioxidants

Ascorbic acid was selected as a representative antioxidant for colorimetric studies. Due to the strong oxidizing properties of oxTMB, AA can make the color of oxTMB lighten or even disappear into being colorless. Therefore, adding different concentrations of AA to the reaction system results in different absorbance values at 652 nm, enabling the establishment of a colorimetric method for the detection of AA. In the reaction system, 0.1 mL of AA with a concentration ranging from 0 to 100 mM was added and reacted for 10 min at room temperature. The absorbance value was measured at 652 nm.

For the actual sample analysis, four kinds of fruits and vegetables were selected, including Chinese cabbage, orange, kiwi, and cabbage. The fruits and vegetables were peeled, beaten into a homogenate, and filtered to obtain a supernatant. Then, they were diluted 50, 100, 200, and 300 times, respectively. Using the same AA detection method, the measured absorbance values were substituted into the standard curve to calculate the concentrations of the four vegetables and fruits. These concentrations were then multiplied by the dilution ratios, and subsequently converted to millimolar equivalents to obtain the TAC values of the actual samples.

## 3. Results and Discussion

### 3.1. Characterization of Cu-TCPP and CuO-TCPP

**Figure 1:**
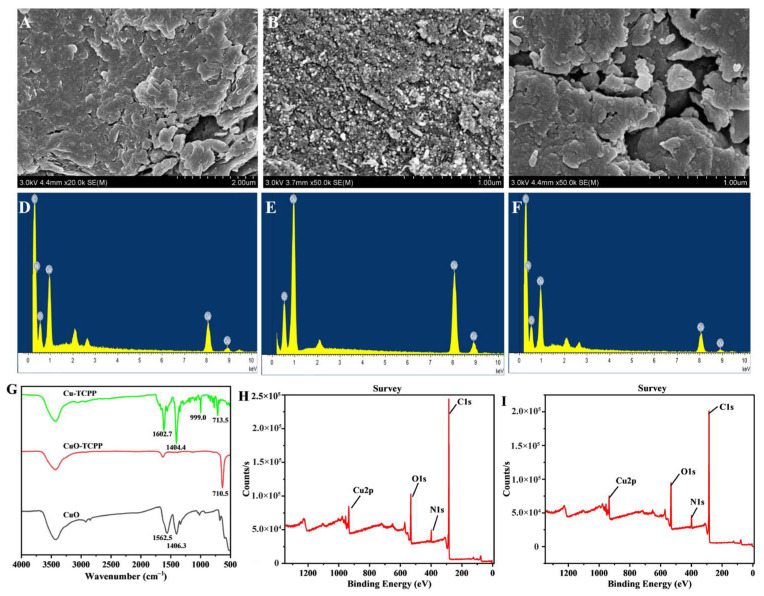
SEM diagram of Cu-TCPP (**A**), CuO (**B**), CuO-TCPP (**C**). Energy spectrum point map of Cu-TCPP (**D**), CuO (**E**), and CuO-TCPP (**F**). (**G**) FTIR of Cu-TCPP, CuO, and CuO-TCPP. XPS characterization of Cu-TCPP (**H**), CuO-TCPP (**I**).

Figure 1A–C present the morphological characterizations of CuO, Cu-TCPP, and CuO-TCPP. The results indicate that CuO exhibits a small granular structure, while Cu-TCPP and CuO-TCPP possess a two-dimensional layered structure. Additionally, CuO deposition was observed on the surface of CuO-TCPP. The energy dispersive spectrometry point measurement graph (Figure 1D–F) demonstrates that CuO exhibits peak and the corresponding peak sizes for the two elements Cu and O. Both Cu-TCPP and CuO-TCPP can be observed to have peaks and corresponding peak sizes for the four elements Cu, O, C, and N, verifying the successful synthesis of Cu-TCPP and CuO-TCPP.

According to the literature [70], the peak of H2-TCPP appears near 1700 cm^−1^, and this peak corresponds to C=O. As shown in Figure 1G, the peak of Cu-TCPP at 1700 cm^−1^ is not very prominent. Instead, new peaks appear at 1612.7 cm^−1^, 1404.4 cm^−1^, and 999.0 cm^−1^, which correspond to the CO-O-Cu bond, benzene ring skeleton, H-O bond, and C=O bond, respectively. This evidence demonstrates the formation of coordination bonds between Cu^2+^ and TCPP. CuO exhibits vibration peaks at 1562 cm^−1^ and 1406 cm^−1^. CuO-TCPP shares two vibration peaks with CuO in the same positions, but the peak intensities of CuO-TCPP are higher than those of CuO. Additionally, CuO-TCPP also exhibits a vibration peak at 710.5 cm^−1^, which corresponds to the H-O bond. These findings indicate the successful synthesis of CuO-TCPP.

**Figure 2:**
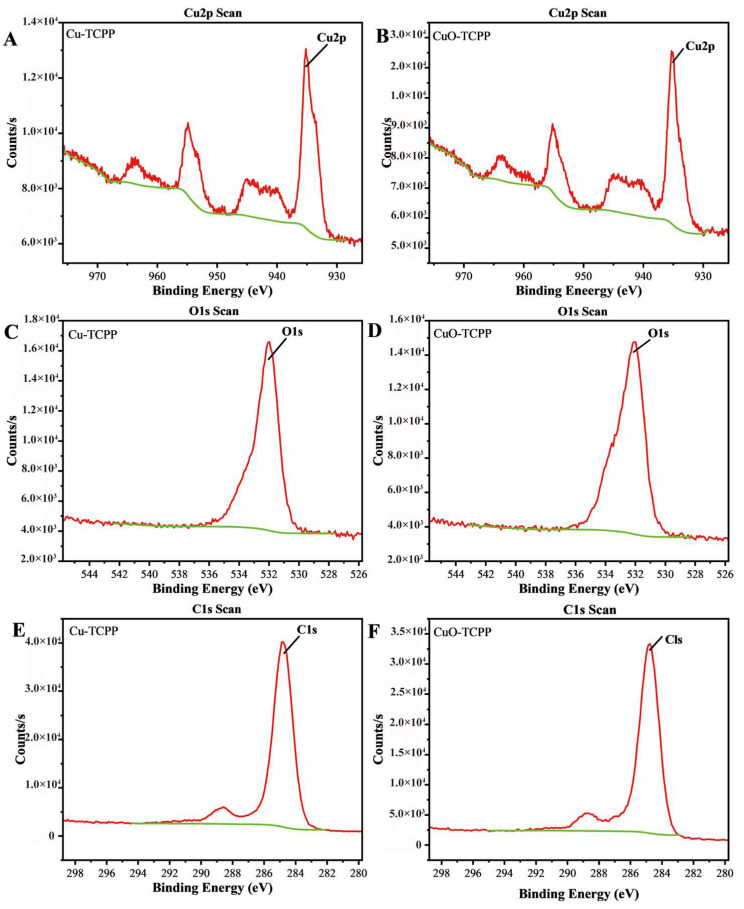
XPS spectra of Cu-TCPP and CuO-TCPP. (**A**) Cu2p of Cu-TCPP, (**B**) Cu2p of CuO-TCPP, (**C**) O1s of Cu-TCPP, (**D**) O1s of CuO-TCPP, (**E**) C1s of Cu-TCPP, and (**F**) C1s of CuO-TCPP.

**Figure 3:**
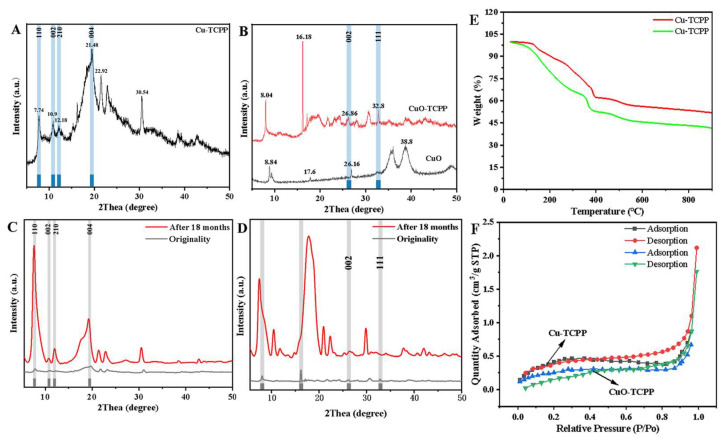
XRD characterization of Cu-TCPP (**A**,**C**) and CuO-TCPP (**B**,**D**). (**E**) TGA of Cu-TCPP and CuO-TCPP. (**F**) BET characterization diagram of Cu-TCPP and CuO-TCPP.

The XPS spectra of Cu-TCPP and CuO-TCPP are shown in Figure 1H,I, and the characteristic peaks of the valence states of Cu, O, N, C and other elements appear. Figure 2 presents the high-resolution spectra of Cu2p, O1s, and C1s spectra. Analysis of the Cu spectra reveals that Cu is present in the +2 oxidation state, with a characteristic peak at 934.3 eV. Regarding the C1s spectra, the peaks at 284.4 eV and 288.3 eV are attributed to C=C and O-C=O bonds. Furthermore, the O1s spectra indicate that the peak at 531.5 eV originates from C-O bonds. Figure 3A,B present the XRD spectra of Cu-TCPP, CuO, and CuO-TCPP. Cu-TCPP exhibits strong diffraction peaks at 2θ = 7.74°, 10.9°, 12.18°, 21.48°, 22.92°, and 30.54°, which is consistent with previous studies [71], indicating the successful synthesis of Cu-TCPP. CuO displays diffraction peaks at 2θ = 8.84°, 17.6°, 26.16°, and 38.8°, while CuO-TCPP exhibits diffraction peaks at 2θ = 8.04°, 16.18°, 26.86°, and 32.8°. The peaks at 26.86° and 32.8° correspond to the crystal planes (002) and crystal planes (111) of CuO. Comparison with the standard card PDFNO.45-0937 reveals that the CuO present in CuO-TCPP exhibits good crystallinity, confirming the successful synthesis of CuO-TCPP.

In addition, in order to verify the stability of Cu-TCPP and CuO-TCPP, the XRD patterns of Cu-TCPP (Figure 3C) and CuO-TCPP (Figure 3D) after 18 months of placement were analyzed and compared with those of the newly synthesized material. The results show that the position of the XRD diffraction peak of Cu-TCPP after 18 months of placement basically corresponds to that of the newly synthesized Cu-TCPP, that is, the crystal structure of Cu-TCPP does not change after 18 months of placement, and the stability is good. However, after 18 months of CuO-TCPP, the position of the XRD diffraction peak changed slightly from the newly synthesized one, the crystal structure changed, and the stability was not as good as that of Cu-TCPP.

The BET characterization results for Cu-TCPP and CuO-TCPP are resented in Figure 3F. When the relative pressure is within the range of 0 < P/P0 < 0.6, the adsorption and desorption rates of both materials increase gradually. However, when the relative pressure falls within 0.8 < P/P0 < 1.0, a sharp increase in adsorption and desorption rates is observed, attributed to the presence of pores with different sizes within the composite materials. The specific surface areas, pore volumes, adsorbed pore diameters, and desorbed pore diameters of Cu-TCPP and CuO-TCPP are 1.6048 m^2^/g and 0.9868 m^2^/g; 0.003063 cm^3^/g and 0.002910 cm^3^/g; 7.7779 nm and 10.5520 nm; and 7.9594 nm and 10.7830 nm, respectively. Therefore, while Cu-TCPP and CuO-TCPP exhibit relatively small specific surface areas, they possess stable structures. Figure 3E presents the TGA plots of Cu-TCPP and CuO-TCPP, which exhibit similar weight loss characteristics. Both materials underwent a slight weight loss between 30 °C and 125 °C, primarily due to the evaporation of water and ethanol present in the samples. A rapid weight loss occurred between 104 °C and 418 °C, likely attributed to the breakage of C=C and C=O bonds. As the temperature rises above 450 °C, the rate of weight loss gradually decreases. At 900 °C, the residual mass fractions of Cu-TCPP and CuO-TCPP are 34.6% and 41.7%, respectively.

### 3.2. Optimization of Experimental Conditions and Steady-State Kinetic

**Figure 4:**
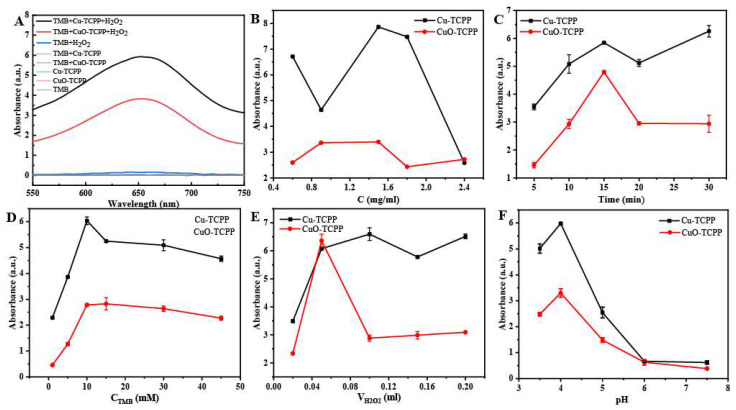
(**A**) Detection of nanozyme activity of Cu-TCPP and CuO-TCPP (under optimal conditions). (**B**) Optimization results of nanozyme concentration. (**C**) Reaction time optimization results. (**D**) TMB concentration optimization results. (**E**) H_2_O_2_ volume optimization results. (**F**) Buffer pH optimization results.

Peroxidase-like activity assays for Cu-TCPP and CuO-TCPP are shown in Figure 4A. From the results, it can be seen that the absorbance value of the system with Cu-MOFs, TMB and H_2_O_2_ is much higher than that of the system without Cu-MOFs. It was proved that Cu-MOFs had peroxidase activity that catalyzed the chromogenic development of H_2_O_2_ oxidation TMB.

To select the optimal experimental conditions for the reaction system, we investigated the effects of nanozyme concentration, reaction time, catalytic substrate TMB concentration, H_2_O_2_ volume, and the pH of the buffer on enzyme activity. A UV-visible spectroscopy method was employed for full-spectrum scanning, and the absorbance of the reaction system was measured at three wavelengths of 645 nm, 650 nm, and 655 nm, respectively. After conducting three parallel tests and selecting the highest value, the average absorbance at the three wavelengths was taken as the experimental result. First, TMB solution (0.1 mL, 15 mM), H_2_O_2_ (0.1 mL, 30%), and acetate buffer solution (3 mL, 10 mM, pH 4) were added to the reaction system. After that, 0.1 mL (0.6–2.4 mg/mL) of nanozyme aqueous solution was added separately, and the absorbance was measured after a reaction for 15 min at room temperature. As shown in Figure 4B, the enzyme activity of these two nanomaterials peaked when their concentrations were set at 1.5 mg/mL. Then, in the aforementioned reaction system, nanozyme aqueous solution at a concentration of 1.5 mg/mL was selected, and the reaction was allowed to proceed for 5, 10, 15, 30, and 45 min at room temperature to measure the absorbance values. The results (Figure 4C) indicate that when the reaction time was 15 min, the peroxidase activity of these two nanomaterials was significant.

Thirdly, in the same reaction system, the TMB solution concentration was set to range from 1 to 45 mM, and nanozyme aqueous solution with a concentration of 1.5 mg/mL was selected. The reaction was allowed to proceed for 15 min at room temperature. As shown in Figure 4D, in the Cu-TCPP reaction system, the absorbance value of the TMB solution was the highest when the concentration was 10 mM. When the concentration of the TMB solution in the CuO-TCPP reaction system was 10 mM and 15 mM, the absorbance value was higher. In order to ensure that there was enough TMB to react with H_2_O_2_, 15 mM TMB solution was selected as the optimal reaction concentration for both Cu-TCPP and CuO-TCPP reaction systems. Finally, based on the above results, the volume of H_2_O_2_ was optimized. In the reaction system, 0.1 mL 15 mM TMB solution was added, along with 0.02 mL, 0.05 mL, 0.1 mL, 0.15 mL, and 0.2 mL of 30% H_2_O_2_, respectively. To ensure the total volume of the reaction system remained unchanged, the volumes of the acetate buffer solution were adjusted to 3.08 mL, 3.05 mL, 3 mL, 2.95 mL, and 2.9 mL accordingly. Then, 0.1 mL of 1.5 mg/mL nanozyme aqueous solution was added, and the reaction was allowed to proceed for 15 min at room temperature. Based on the experimental results (Figure 4E), 0.05 mL of H_2_O_2_ was selected as the optimal reaction volume.

Finally, the optimal pH of the reaction system was optimized. Buffers with pH 3.5, 4, 5, 6, and 7.5 were selected, and the absorbance values were detected after 0.1 mL 1.5 mg/mL Cu-TCPP or CuO-TCPP, 0.1 mL 10 mM TMB, and 0.05 mL 30% H_2_O_2_ for 15 min. The results are shown in Figure 4F. The optimal pH for Cu-TCPP and CuO-TCPP is 4, both Cu-TCPP and CuO-TCPP had good catalytic activity under acidic conditions, and the catalytic activity became weaker when the pH value was greater than 5. Therefore, the optimal experimental conditions for Cu-TCPP and CuO-TCPP are as follows: the pH of the buffer is 4, the material concentration is 1.5 mg/mL, the hydrogen peroxide volume is 0.05 mL, the TMB concentration is 15 mM, and the reaction time is 15 min.

**Figure 5:**
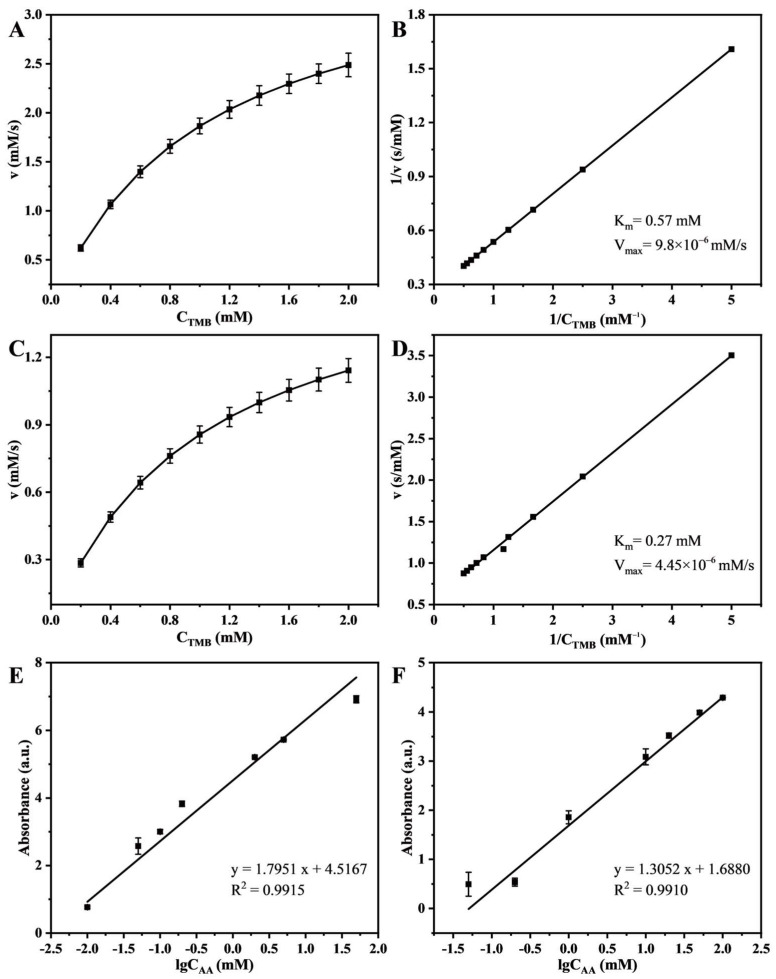
Kinetic analysis of Cu-TCPP (**A**,**B**) and CuO-TCPP (**C**,**D**). Cu-TCPP (**E**) and CuO-TCPP (**F**) are the linear relationship between the lg10 of AA concentration and absorbance value.

Under the optimal reaction conditions mentioned above, steady-state kinetic analysis was performed on Cu-TCPP and CuO-TCPP to calculate the kinetic parameters of the enzymes (Figure 5A–D). The results showed that the K_m_ value of Cu-TCPP was 0.27 mM, and the V_max_ was 4.45 × 10^−6^ M/s. For CuO-TCPP, the K_m_ value was 0.57 mM, and the V_max_ was 9.8 × 10^−6^ M/s. K_m_ is an important parameter for evaluating the affinity between enzymes and substrates. A lower K_m_ value indicates better interaction between the nanoenzyme and the substrate.

### 3.3. Detection of Antioxidant

Antioxidants can inhibit the oxidation reaction based on peroxidase activity to a certain extent, that is, oxTMB can be reduced to TMB, making the solution lighter or even colorless. In this study, AA with a concentration range of 0–100 mM was selected as an antioxidant for the colorimetric assay. The results demonstrated that there was a good linear relationship between the absorbance at 652 nm and the lgC_AA_ ranging from −2 to 2 (Cu-TCPP) and −1.3 to 2 (CuO-TCPP), as was the case for the concentration of AA ranging from 0.01 to 100 mM (Cu-TCPP) and 0.05–100 mM (CuO-TCPP), verifying the feasibility of the detection method. Due to the large difference in concentration range, the log10 of AA concentration was taken as the *x*-axis, and the difference between the absorbance at 0 mM AA concentration and the absorbance at other concentrations was used as the *y*-axis. Figure 5E,F show the linear relationship between AA and absorbance under two different nanozyme catalysis conditions. The peroxidase-like activity and linear range of detection of Cu-TCPP and CuO-TCPP were compared with other typical nanozymes, as shown in Table 1. Obviously, the linear range of detection of Cu-TCPP and CuO-TCPP for AA is much higher than that reported in other articles, but the affinity for TMB (the smaller the Km value, the greater the affinity) is among the other nanozymes. Figure 5E represents the catalytic reaction of Cu-TCPP, and the linear equation for the measured data is y = 1.7951x + 4.5167, R^2^ = 0.9915. Figure 5F demonstrates the catalytic reaction of CuO-TCPP, and the equation is y = 1.3052x + 1.6880, R^2^ = 0.9910.

**Figure 6:**
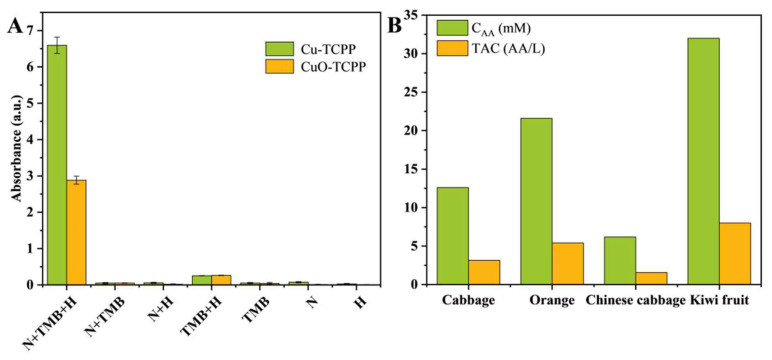
(**A**) Peroxidase-mimicking activity of Cu-TCPP and CuO-TCPP, and the UV-Vis absorption spectra of Cu-TCPP and CuO-TCPP in different substrates (N: Cu-TCPP or CuO-TCPP nanozyme, H: H_2_O_2_, 652 nm). (**B**) AA concentration and total antioxidant capacity test results of eight actual samples.

In addition, the peroxidase-like activities of Cu-TCPP and CuO-TCPP were verified. As shown in Figure 6A, it can be observed that only the nanozyme-TMB-H_2_O_2_ system showed an obvious blue color, indicating that Cu-TCPP and CuO-TCPP can catalyze the oxidation of TMB by H_2_O_2_. Moreover, the control experiments showed that the absorbance of the nanozyme-TMB-H_2_O_2_ system at 652 nm was much higher than that of the TMB-H_2_O_2_, nanozyme-TMB, and other systems. These results can confirm that Cu-TCPP and CuO-TCPP have peroxidase-like activities.

### 3.4. Detection of AA Concentration and TAC in Actual Samples

Based on the experimental method, Cu-TCPP and CuO-TCPP were separately used to detect the AA concentration and TAC of four types of fruits and vegetables. Each nanozyme was applied to determine the AA concentration and TAC of two different fruits and vegetables through the comparison of RSD. The detection results are shown in Figure 6B. Cu-TCPP was used to detect cabbage and oranges, with the AA concentration of cabbage being 15.45 mM, with a TAC of 3.86 AA/L and RSD of 2.89%. The AA concentration of oranges was 22.5 mM, with a TAC of 5.63 AA/L and RSD of 1.69%. CuO-TCPP was used to detect Chinese cabbage and kiwi fruits, with the AA concentration of Chinese cabbage being 6.2 mM, with a TAC of 1.55 AA/L and RSD of 2.02%. The AA concentration of kiwi fruits was 32 mM, with a TAC of 8 AA/L and RSD of 0.6%. The total antioxidant capacity of the selected fruits was generally higher than that of vegetables. Cu-TCPP and CuO-TCPP can be utilized for detecting the TAC of fruits and vegetables. Through the detection of TAC, it is possible to distinguish the levels of antioxidant substances in fruits and vegetables, thus potentially providing assistance to people in terms of healthy dietary choices.

## 4. Conclusions

In this study, two 2D, layered, copper-based MOFs nanozymes (Cu-TCPP and CuO-TCPP) were synthesized via an in situ polymerization method using TCPP, Cu^2+^, and CuO to enhance their peroxidase activity. Cu-TCPP and CuO-TCPP can catalyze the oxidation of colorless TMB to blue oxTMB by H_2_O_2_. After adding antioxidants to the above solution, oxTMB was reduced to TMB, resulting in a lighter blue color and decreased absorbance. Selecting AA as a representative antioxidant, a convenient and rapid colorimetric sensing method was developed to determine TAC using the peroxidase-like activity of Cu-TCPP and CuO-TCPP. This method was successfully applied to the measurement of TAC in fruits and vegetables. The results were generally consistent with those reported previously [78], and Cu-TCPP and CuO-TCPP exhibited a broad detection range at 652 nm and a high affinity for the substrate. This study not only provides a novel method for preparing nanozymes with peroxidase-like activity but also offers a simple analytical approach for detecting TAC in fruits and vegetables.

## Figures and Tables

**Table 1:** Comparison of peroxidase-like activity and linear range of detection of Cu-TCPP and CuO-TCPP with other typical nanozymes.

Nanozyme	Substrate	Km (mM)	Linear Range (µM)	Ref.
Cu-TCPP	TMB	0.57	10~1 × 10^5^	This work
CuO-TCPP	TMB	0.27	50~1 × 10^5^	This work
Cu-SAC	TMB	0.077	1~30	[72]
Au@Cu-HCF	TMB	-	7~250	[73]
CuBi aerogel	TMB	2.7	300~900	[60]
Cu2-xSe	TMB	0.457	0.5~50	[63]
PdCoOx	TMB	0.16	0~700	[74]
Fe3+-PA	TMB	1.15	2~40	[75]
UoZ-4	TMB	0.071	3~25	[76]
CDs	TMB	-	1~30	[77]

## Data Availability

The original contributions presented in the study are included in the article, further inquiries can be directed to the corresponding author.
